# A study on Internet use and subjective well-being among Chinese older adults: based on CGSS (2012-2018) five-wave mixed interface survey data

**DOI:** 10.3389/fpubh.2023.1277789

**Published:** 2024-01-05

**Authors:** Chong Zhang, Yong Zhang, Yan Wang

**Affiliations:** ^1^Institute of Network Social Governance, School of Marxism, University of Electronic Science and Technology of China, Chengdu, China; ^2^School of Economics and Trade, Guangdong University of Finance, Guangzhou, China; ^3^School of Law and Sociology, Xihua University, Chengdu, China

**Keywords:** internet use, subjective well-being, propensity score matching, subjective social fairness, mediation effect, older adults

## Abstract

**Objective:**

This study is designed to investigate the relationship between Internet use and subjective well-being of the older adults in China, and to analyze the mediating role of subjective social fairness in the above relationship.

**Methods:**

Based on the five-wave mixed interface survey data of China General Social Survey (CGSS) in 2012, 2013, 2015, 2017, and 2018, we select a total of 18,458 older adults aged 60 and above, and comprehensively used ordered probit regression, propensity score matching (PSM), and Karlson-Holm-Breen (KHB) mediating effect test methods.

**Results:**

The analysis results show that (1) Internet use is significantly positively correlated with the subjective well-being of the older adults, and the higher the frequency of use, the stronger the subjective well-being. (2) Place of residence, education, and regional factors moderated the effect of Internet use on subjective well-being. The subjective well-being effect of Internet use is significant among male older adults, as well as on urban, educated, or eastern older adults. (3) Subjective social fairness plays a negative mediating role in the relationship between Internet use and subjective well-being of older adults.

**Conclusion:**

The findings suggest that Internet use contributes to the improvement of subjective well-being in older adults, but attenuates this effect by reducing the subjective social fairness. Future research should further consider other factors such as the purpose, specific function, intensity of Internet use, etc., to gain a deeper understanding of how the Internet can help promote well-being.

## 1 Introduction

With the development of the digital age, the Internet has become an important tool and main carrier in the life of the older adults in China, which is related to the daily life, mental health, and even well-being of the older adults. However, there is still no consensus in the academic community on the impact of Internet use on well-being of older adults. The research on well-being began in developed countries in Europe and the United States in the 1950s. In the 1970s, the American economist Easterlin proposed the famous “Easterlin paradox” (1). After that, the research on well-being has increasingly become a theoretical hot spot and a practical focus of attention in academia (2, 3). Subjective well-being (SWB) is described as the cognitive judgments and emotional states regarding an individual’s quality of life (4). SWB is also referred to as “life satisfaction” and “happiness” (5). With the rapid development of the Internet after the 1990s, the research on Internet use and subjective well-being has gradually become an important topic in the fields of psychology, economics, sociology, and political science. Summarizing the previous literature, it can be found that there are two diametrically opposed views. Some scholars believe that Internet use has a significant positive impact on subjective well-being (6). Using the Internet can reduce loneliness (7), improve communication with friends and relatives (8), gain more social support (9, 10), and may even help improve income (11). Therefore, residents with higher frequency of Internet use have stronger subjective well-being (12). Other scholars believe that Internet use has a significant negative impact on subjective well-being (13). The Internet may lead to a decrease in people’s social connections (14), a decrease in social trust (15), and even a comparison between incomes (16), thereby reducing subjective well-being. Especially adolescents are prone to lose themselves due to Internet addiction and have a lower well-being (17, 18).

Although there is a lot of literature on the effects of Internet use on subjective well-being, why are there so different conclusions? It may be due to the large differences between different age groups and different countries, as well as the different degrees of Internet penetration. For example, the frequent Internet access of teenagers is often not conducive to their subjective well-being. Looking back at previous studies, few scholars have focused on the Chinese older adults. The research results of some scholars show that Internet use can improve the well-being of the older adults (19, 20). Compared with the older adults who do not use the Internet, the older adults who use the Internet can enhance social interaction (21), obtaining social support (9), reducing loneliness (3, 22), improving intergenerational relationships (2), and improving subjective well-being (23, 24). However, there are also studies showing that Internet use has no significant effect on the well-being of the older adults (25).

With the completion of China’s comprehensive well-off society, after meeting material needs, the older adults hope to improve their well-being through higher pursuits. According to Maslow’s hierarchy of needs, human needs can be divided into five levels: survival, safety, social, esteem and self-actualization. The use of the Internet enhances the social, esteem, and self-actualization needs of older adults and is an important manifestation of active ageing. Active ageing emphasizes social interaction and social participation, and the use of the Internet can help the older adults to better communicate and participate, and thus obtain more social support. Activity theory also suggests that life satisfaction in older adults is positively related to daily activities, and although older adults retire from work, they can look for other alternatives to work, replacing old friends with people in new environments. Therefore, the activity theory advocates that the depression caused by the interruption of social roles in the older adults can be improved through new participation and new roles. Using the Internet after retirement is a good substitute, which can not only keep in touch with old friends, but also meet new friends, expand social contacts, and obtain more social support. Therefore, the use of the Internet should help improve the well-being of old age. However, if you only use the Internet occasionally or rarely, the effect may not be obvious. Some scholars have found that whether the use of the Internet has no significant impact on residents’ subjective well-being (26). However, considering that the proportion of the older adults who never use the Internet is relatively high, and the older adults who use the Internet will be different, it is inferred that the older adults who use the Internet will have a relatively high well-being, and the well-being will rise as the frequency of Internet use increases, and older adults who use the Internet regularly are happier. Accordingly, this study proposes the first set of hypotheses:

*Hypothesis 1*: The higher the frequency of Internet use, the stronger the subjective well-being of older adults.

*Hypothesis 1a*: Compared to the older adults who never use the Internet, the older adults who use the Internet have a stronger subjective well-being.

*Hypothesis 1b*: Compared to the older adults who do not frequently use the Internet, the older adults who frequently use the Internet have a stronger subjective well-being.

In addition, due to the existence of objective phenomena such as the “digital divide” brought about by the urban–rural dual structure and unbalanced regional development, the Internet usage of the older adults in different regions is quite different (27). People’s physiological conditions and cognitive abilities are also different, so the well-being effect of the Internet may also be different. Therefore, the effect of Internet use on the subjective well-being of older adults’ groups with different characteristics is heterogeneous. Thus, a second set of hypotheses is proposed:

*Hypothesis 2*: There is heterogeneity in the impact of Internet use on the subjective well-being of the older adults.

*Hypothesis 2a*: There are gender differences in the effect of Internet use on the subjective well-being of the older adults.

*Hypothesis 2b*: There are urban and rural differences in the effect of Internet use on the subjective well-being of the older adults.

*Hypothesis 2c*: There are educational differences in the effect of Internet use on the subjective well-being of the older adults.

*Hypothesis 2d*: There are regional differences in the effect of Internet use on the subjective well-being among older adults.

From the previous literature, the impact of Internet use on well-being is often through some positive mediating variables (3, 9, 28). But does the Internet also counteract its effects on well-being through some negative factors? Especially in the study of the older adults, why there are some positive and significant effects, while others are not. Many previous studies have shown that Network media will depict social facts through symbols such as words, pictures, sounds, and images, which directly affect people’s construction of subjective social facts (29). In addition, its specific reports shape people’s cognition and understanding of economic, political, health, technology, and social risks (30, 31). People’s comparisons, conformity, imitation and other psychology are greatly influenced by network information (32, 33). If some media cannot objectively and rationally evaluate the social status quo, it will also cause the people’s subjective social fairness to deviate from the actual situation (34). As a kind of psychological perception, subjective social fairness has an important influence on well-being. People are unhappy because perceive their lower relative economic and relative status in the social group (35, 36). Therefore, this study chose subjective social fairness as a mediating variable to more scientifically explore the impact of Internet use on the subjective well-being of the older adults. From this, a third set of hypotheses is proposed:

*Hypothesis 3*: Subjective social fairness plays a mediating role in the effect of the Internet use on the subjective well-being of the older adults.

*Hypothesis 3a*: Internet use reduces older adults’ subjective social fairness.

*Hypothesis 3b*: Subjective social fairness has a significant positive effect on the subjective well-being of the older adults.

*Hypothesis 3c*: Subjective social fairness plays a negative mediating role in the effect of Internet use on the subjective well-being of the older adults.

## 2 Methods

### 2.1 Data and sample

The data comes from the Chinese General Social Survey (CGSS) which began in 2003 and was initiated by the National Survey Research Center at Renmin University of China (NSRC). It is the earliest nationwide, comprehensive, and continuous large-scale social survey project in China. The survey uses multi-level stratified PPS random sampling to conduct a continuous cross-sectional survey of more than 10,000 households in 28 provinces, autonomous regions, and municipalities in mainland China, systematically and comprehensively collects data at multiple levels of society, community, family, and individual. Based on the research content and research needs, this study selects the five-phase mixed cross-section data in 2012, 2013, 2015, 2017, and 2018 that are closest to the current time. The research object is the older adults aged 60 and above. After excluding some missing and invalid samples, a total of 18,458 valid samples were obtained, of which 3,198, 3,083, 3,415, 4,240 and 4,522 samples were in 2012, 2013, 2015, 2017, and 2018, respectively.

### 2.2 Measures

#### 2.2.1 Outcome variable

The measure of subjective well-being is based on the question “In general, do you think your life is happy?” in CGSS. The respondents answered that the answers included “very unhappy,” “relatively unhappy,” “neither happy nor unhappy,” “relatively happy,” and “very happy.” The above answers were assigned as 1, 2, 3, 4, and 5 in turn.

#### 2.2.2 Explanatory variables

The measure of Internet use is based on the question “In the past year, how did you use the following media?,” the Internet (including mobile Internet access) was one of the items, and the answers were “never, rarely, sometimes, often, very frequently,” assigned as 1, 2, 3, 4, 5 in turn. In addition to the frequency of Internet use, the Internet use behavior of older adults can also be measured by whether they use the Internet, whether they frequently use the Internet, and whether they frequently use the Internet during free time. Whether to use the Internet and whether to frequently use the Internet are also measured through the previous questions and options. If the older adults choose “never,” it means they do not use the Internet; if they choose the other four options, it means they use the Internet; If they select “often” or “very frequently,” it indicates frequent use of the Internet, while vice versa, it indicates infrequent use of the internet. Whether to frequently use the Internet during free time is based on the question “Have you often engaged in the following activities in your free time in the past year?” The options are “never,” “several times a year or less,” “several times a month,” “several times a week,” and “every day,” if they choose “several times a month” or “several times a week,” it indicates frequent use of the Internet during free time.

#### 2.2.3 Mediating variable

Subjective social fairness comes from the question “In general, do you think today’s society is fair?” The answers are “completely unfair, relatively unfair, neither fair nor unfair, relatively fair, and completely fair,” and assign the values to 1, 2, 3, 4, and 5 in turn.

#### 2.2.4 Covariates

Referring to existing research, this study selects variables such as individual characteristics, family characteristics, social characteristics and regions as covariates. Individual characteristics include gender (male = 1, female = 0), age (60 ~ 118), place of residence (urban = 1, rural = 0), education level (schooled = 1, unschooled = 0), marital status (with a spouse = 1, without a spouse = 0), health status. Among them, the health question comes from the item in questionnaire “In the past four weeks, how often did health problems affect your work or other daily activities? “, the respondents answer “always” or “often” is classified as unhealthy and assigned a value of 0, and the others are classified as healthy and assigned a value of 1. Family characteristics include the population living together (1~21), the number of sons (0 ~ 8), and the number of daughters (0 ~ 12). Social characteristics including social class and social interaction, are, respectively, from the items. “In general, in the current society, which level of society do you belong to? (1–10 points, the highest 10 points represent the most top level, with the lowest 1 being the bottom)” and “In the past year, how often did you do the following in your free time (social/visit, never, rarely, sometimes, often, very often)?.” According to the division standard of the 2006 China Statistical Yearbook for the three major regions of the eastern, central and western parts of mainland China, the 31 provinces and cities in the mainland are divided into eastern, central and western regions.

### 2.3 Data analysis

Based on the data of 2012, 2013, 2015, 2017 and 2018 of CGSS, we first use the ordered probit model to study the effect of the frequency of Internet use on the subjective well-being of the older adults, and take whether to use the Internet, whether to frequently use the Internet, and whether to frequently use the Internet during free time as substitute variables to test the robustness of the above relationship. Secondly, we use propensity score matching (PSM) to eliminate the selection bias of the sample (37, 38), to further test whether the Internet use affects the subjective well-being of the older adults, while also dividing the sample into different gender, place of residence, education level, and region for heterogeneity analysis. Finally, the mediating effect of subjective social fairness in the relationship between Internet use and well-being of the older adults was investigated by stepwise test and KHB method (39).

## 3 Result

### 3.1 Descriptive statistics

Table 1 shows the characteristics of the sample. Overall, the average age of the sample is 69 years old. The proportion of male older adults is slightly lower than that of female older adults; the proportion of urban older adults is higher than that of rural older adults; the proportion of educated older adults is much higher than that of uneducated older adults; the proportion of eastern older adults is higher than that of central and western older adults. Most older adults never use the Internet, and only about 10% of older adults report that they use the Internet regularly, but it is worth mentioning that the proportion that use the Internet shows an upward trend, while the proportion of those who never or rarely use it is declining. At the same time, nearly 80% of older adults report they are overall happy, and the proportion of happy is also showing an upward trend.

**Table 1:** Sample characteristics.

Variables	2012	2013	2015	2017	2018	Total
Subjective well-being, *N* (%)						
Very unhappy	43 (1.34)	55 (1.78)	42 (1.23)	59 (1.39)	54 (1.19)	253 (1.37)
Relatively unhappy	230 (7.19)	231 (7.49)	216 (6.33)	283 (6.67)	275 (6.08)	1,235 (6.69)
Neither happy nor unhappy	446 (13.95)	543 (17.61)	427 (12.5)	524 (12.36)	513 (11.34)	2,453 (13.29)
Relatively happy	1895 (59.26)	1755 (56.93)	2012 (58.92)	2,461 (58.04)	2,646 (58.51)	10,769 (58.34)
Very happy	584 (18.26)	499 (16.19)	718 (21.02)	913 (21.53)	1,034 (22.87)	3,748 (20.31)
Frequency of Internet use, *N* (%)						
Never	2,917 (91.21)	2,782 (90.24)	2,903 (85.01)	3,252 (76.7)	3,191 (70.57)	15,045 (81.51)
Rarely	90 (2.81)	127 (4.12)	182 (5.33)	215 (5.07)	298 (6.59)	912 (4.94)
Sometimes	60 (1.88)	68 (2.21)	116 (3.40)	175 (4.13)	288 (6.37)	707 (3.83)
Often	60 (1.88)	50 (1.62)	109 (3.19)	311 (7.33)	399 (8.82)	929 (5.03)
Very often	71 (2.22)	56 (1.82)	105 (3.07)	287 (6.77)	346 (7.65)	865 (4.69)
Using the Internet frequently during free time, *N* (%)						
Yes	3,024 (94.56)	2,946 (95.56)	3,136 (91.83)	3,457 (81.53)	3,506 (77.53)	16,069 (87.06)
No	174 (5.44)	137 (4.44)	279 (8.17)	783 (18.47)	1,016 (22.47)	2,389 (12.94)
Subjective social fairness, *N* (%)						
Totally unfair	170 (5.32)	172 (5.58)	105 (3.07)	257 (6.06)	239 (5.29)	943 (5.11)
Somewhat unfair	650 (20.33)	701 (22.74)	588 (17.22)	887 (20.92)	847 (18.73)	3,673 (19.90)
Neither fair nor unfair	611 (19.11)	682 (22.12)	665 (19.47)	718 (16.93)	899 (19.88)	3,575 (19.37)
Relatively fair	1,558 (48.72)	1,374 (44.57)	1872 (54.82)	2,124 (50.09)	2,263 (50.04)	9,191 (49.79)
Totally fair	209 (6.54)	154 (5.00)	185 (5.42)	254 (5.99)	274 (6.06)	1,076 (5.83)
Gender, *N* (%)						
Male	1745 (54.56)	1,590 (51.57)	1,639 (47.99)	2067 (48.75)	2,144 (47.41)	9,185 (49.76)
Female	1,453 (45.43)	1,493 (48.43)	1776 (52.01)	2,173 (51.25)	2,378 (52.59)	9,273 (50.24)
Age, Mean (SD)	69.22 (7.30)	69.02 (7.21)	69.50 (7.51)	69.32 (7.36)	69.58 (7.42)	69.35 (7.37)
Place of residence, *N* (%)						
Rural	1,454 (45.47)	1,388 (45.02)	1,551 (45.42)	1731 (40.83)	1,491 (32.97)	7,615 (41.26)
Urban	1744 (54.53)	1,695 (54.98)	1864 (54.58)	2,509 (59.17)	3,031 (67.03)	10,843 (58.74)
Education level, *N* (%)						
Unschooled	999 (31.24)	1,047 (33.96)	1,023 (29.96)	1,069 (25.21)	1,272 (28.13)	5,410 (29.31)
Schooled	2,199 (68.76)	2036 (66.04)	2,392 (70.04)	3,171 (74.79)	3,250 (71.87)	13,048 (70.69)
Marital status, *N* (%)						
Without a spouse	911 (28.49)	832 (26.99)	870 (25.48)	1,134 (26.75)	1,212 (26.80)	4,959 (26.87)
With a spouse	2,287 (71.51)	2,251 (73.01)	2,545 (74.52)	3,106 (73.25)	3,310 (73.20)	13,499 (73.13)
Physical condition, *N* (%)						
Unhealthy	834 (26.08)	696 (22.58)	748 (21.90)	1,055 (24.88)	954 (21.10)	4,287 (23.23)
Healthy	2,364 (73.92)	2,387 (77.42)	2,667 (78.10)	3,185 (75.12)	3,568 (78.90)	14,171 (76.77)
Population living together, Mean (SD)	2.74 (1.54)	2.68 (1.51)	2.48 (1.35)	2.44 (1.40)	2.44 (1.40)	2.54 (1.44)
Number of sons, Mean (SD)	1.57 (1.09)	1.51 (1.04)	1.40 (1.04)	1.29 (0.96)	1.25 (0.94)	1.39 (1.02)
Number of daughters, Mean (SD)	1.37 (1.22)	1.26 (1.13)	1.20 (1.12)	1.13 (1.09)	1.08 (1.08)	1.19 (1.13)
Social class, Mean (SD)	4.13 (1.73)	4.18 (1.74)	4.33 (1.65)	4.06 (1.76)	4.16 (1.73)	4.17 (1.73)
Social interaction, *N* (%)						
Never	557 (17.42)	332 (10.77)	449 (13.15)	670 (15.80)	667 (14.75)	2,675 (14.49)
Rarely	1,196 (37.40)	960 (31.14)	1,083 (31.71)	1,419 (33.47)	1,494 (33.04)	6,152 (33.33)
Sometimes	811 (25.36)	909 (29.48)	913 (26.73)	1,056 (24.91)	1,119 (24.75)	4,808 (26.05)
Often	522 (16.32)	715 (23.19)	787 (23.05)	837 (19.74)	932 (20.61)	3,793 (20.55)
Very often	112 (3.50)	167 (5.42)	183 (5.36)	258 (6.08)	310 (6.86)	1,030 (5.58)
Region, *N* (%)						
Eastern region	1,270 (39.71)	1,149 (76.77)	1,324 (38.77)	1903 (44.88)	2,102 (46.48)	7,748 (41.98)
Central region	1,133 (35.43)	1,218 (39.51)	1,216 (35.61)	1,385 (32.67)	1,473 (32.57)	6,425 (34.81)
Western region	795 (24.86)	716 (23.22)	875 (25.62)	952 (22.45)	947 (20.94)	4,285 (23.21)
*N*	3,198	3,083	3,415	4,240	4,522	18,458

### 3.2 The relationship between frequency of internet use and subjective well-being

As shown in Table 2, Models 1 to 4 show the changes in the influence of Internet use frequency and subjective social fairness on the subjective well-being of the older adults after successively adding covariates. From Model 1 to 4, the goodness of fit of the model is continuously optimized. *Hypothesis 1* is supported. The frequency of Internet use has a significant positive effect on the subjective well-being of the older adults, that is, the higher the frequency of Internet use, the stronger the subjective well-being of the older adults. *Hypothesis 3b* is supported. Subjective social fairness has a significant positive impact on subjective well-being. The stronger the subjective social fairness, the stronger the subjective well-being of the older adults.

**Table 2:** Ordered probit regression analysis of Internet use and subjective well-being.

Variables	Model 1	Model 2	Model 3	Model 4
Frequency of Internet use	0.060(0.008) ^***^	0.067(0.008) ^***^	0.048(0.008) ^***^	0.038(0.008) ^***^
Subjective social fairness	0.381(0.008) ^***^	0.380(0.008) ^***^	0.344(0.009) ^***^	0.347(0.009) ^***^
Gender	−0.149(0.017) ^***^	−0.144(0.017) ^***^	−0.120(0.018) ^***^	−0.111(0.018) ^***^
Age	0.014(0.001) ^***^	0.013(0.001) ^***^	0.011(0.001) ^***^	0.010(0.001) ^***^
Place of residence	0.119(0.018) ^***^	0.136(0.019) ^***^	0.099(0.019) ^***^	0.063(0.020) ^***^
Education level	0.201(0.020) ^***^	0.210(0.020) ^***^	0.157(0.020) ^***^	0.148(0.020) ^***^
Marital status	0.202(0.020) ^***^	0.178(0.020) ^***^	0.150(0.021) ^***^	0.148(0.021) ^***^
Physical condition	0.376(0.020) ^***^	0.381(0.020) ^***^	0.293(0.020) ^***^	0.285(0.020) ^***^
Population living together		0.026(0.006) ^***^	0.020(0.006) ^***^	0.022(0.006) ^***^
Number of sons		0.023(0.009) ^*^	0.016(0.009)	0.029(0.009) ^***^
Number of daughters		0.029(0.008) ^***^	0.027(0.008) ^***^	0.035(0.008) ^***^
Social class			0.160(0.005) ^***^	0.158(0.005) ^***^
Social interaction			0.066(0.008) ^***^	0.069(0.008) ^***^
Eastern region				0.170(0.021) ^***^
Western region				0.044(0.022) ^*^
Pseudo R^2^	0.069	0.070	0.096	0.098
N	18,458	18,458	18,458	18,458

*p < 0.05, **p < 0.01, ***p < 0.001.

In terms of personal characteristics, older males are less happy than older females. Age has a significant positive impact on the well-being, that is, the older the older adults, the stronger the well-being. Compared with the rural older adults, the urban older adults are happier. Older adults who have attended school are happier than those who have not attended school. Older adults with a spouse are happier than those without a spouse. Healthy seniors are happier than unhealthy seniors. From the perspective of family characteristics, the more people living together, the stronger the subjective well-being among older adults. Older adults with more daughters have stronger well-being, but the number of sons has no significant effect on well-being of the older adults. From the perspective of social characteristics, the older adults with higher social class have stronger subjective well-being. Older adults with higher frequency of social interaction are happier. From the perspective of regional factors, compared with the older adults in the central region, the older adults in the east and west are happier.

### 3.3 Robustness check

In addition to the frequency of Internet use, further select whether to use the Internet, whether to frequently use the Internet, and whether to frequently use the Internet during free time as substitute variables for the frequency of Internet use and Model 5, Model 6 and Model 7 are established, respectively, for robustness test. As shown in Table 3, the results of Model 5 show that there is a significant correlation between using the Internet and subjective well-being. Compared with never using the Internet, the older adults who use the Internet are relatively happier. From Model 6, using the Internet frequently has a significant correlation with subjective well-being, and the older adults who frequently use the Internet are happier than those who do not. The results of Model 7 show that older adults who use the Internet frequently during free time are happier than those who do not. Based on the above results, hypotheses 1a and 1b are supported, that is, the subjective well-being of older adults who use the internet is stronger than those who do not use it; older adults who frequently use the internet have a stronger subjective well-being than those who do not frequently use it.

**Table 3:** Robustness check.

Variables	Model 5	Model 6	Model 7
Using the Internet	0.054(0.023) ^*^		
Using the Internet frequently		0.157(0.030) ^***^	
Using the Internet frequently during free time			0.127(0.027) ^***^
Control covariates	YES	YES	YES
Pseudo R^2^	0.097	0.098	0.098
N	18,458	18,458	18,458

*p < 0.05, **p < 0.01, ***p < 0.001.

### 3.4 PSM analysis of internet Use and subjective well-being

Due to the endogeneity problem caused by the possible sample selection bias between Internet use and individual subjective well-being, the results may be biased if only ordered probit regression analysis is used. To this end, continue to use PSM to estimate and test the processing effect of Internet use. In the selection of specific matching methods, in order to ensure the robustness of the measurement results, our research selects three matching methods: k-nearest neighbor matching, caliper matching, and kernel matching.

Table 4 reports the PSM estimation results of Internet use and well-being. The estimated results show that after removing the sample selection bias, the effect of using the Internet on well-being is still significant. Specifically, after using k-nearest neighbor matching, caliper matching and kernel matching to control for sample heterogeneity between the two groups, the effect of using the Internet on the well-being of the older adults is between 0.070 and 0.072, which further supports *hypothesis 1a*, using the Internet can significantly improve the subjective well-being of the older adults.

**Table 4:** PSM estimation results of Internet use and subjective well-being.

Matching method	Sample	*ATT*	S.E.	T-stat
nearest neighbor matching (k = 4)	Unmatched	0.115	0.016	7.150
	Matched	0.072	0.020	3.570
Caliper matching	Unmatched	0.115	0.016	7.150
	Matched	0.070	0.019	3.760
Nuclear matching	Unmatched	0.115	0.016	7.150
	Matched	0.071	0.019	3.820

ATT represents the Average Treatment Effect on the Treated, which evaluates the average effect of treatment among individuals receiving treatment. It is calculated by comparing the results of the treatment group with those of the control group. T-stat is an indicator used to test whether variables in statistical models have a significant impact on results. If T-stat is greater than 1.96, it indicates significance at the 5% level, and if it is greater than 2.58, it indicates significance at the 1% level.

To ensure the quality of the match and the reliability of the estimated results, a balance check is required. First, from the change of the standardized deviation of each covariate, as shown in Table 5, after matching, the standardized deviation of each covariate is greatly reduced, indicating that the difference between the treatment group (Netizens) and the control group (Non netizens) was reduced. Second, judging from the difference in the mean of covariates, the t-statistic has been greatly reduced after matching, and the mean difference of each covariate is not significant, indicating that the matching effect is very good. Third, the *p*-values in the LR test were all 0.000 before matching, indicating that there were significant differences to varying degrees between the treatment group and the control group before matching, and the p-values after matching were all greater than 0.1, which indicated that there was no significant difference in matching variables between the treatment group and the control group after matching, which minimized sample selection bias. In order to ensure the quality of matching, a joint support hypothesis test is also required. The test results show that 99.9% of the sample observations are matched, and most of the observations are within the common value range, so the common support hypothesis is satisfied. Only part of the test results is given here, and the rest are not reported (other results are generally consistent). If necessary, you can ask the author for it.

**Table 5:** Covariates balance testing.

Variable	Sample	Mean		Bias (%)	Reduct |bias| (%)	t-test	
		T	C			t	*p* > t
Subjective social fairness	U	3.062	3.371	−30.200		−16.120	0.000
	M	3.062	3.051	1.100	96.400	0.440	0.662
Gender	U	0.542	0.488	10.900		5.750	0.000
	M	0.542	0.541	0.200	98.100	0.090	0.931
Age	U	66.771	69.933	−46.200		−22.960	0.000
	M	66.769	66.765	0.100	99.900	0.030	0.978
Place of residence	U	0.891	0.519	89.600		41.780	0.000
	M	0.892	0.881	2.600	97.100	1.420	0.155
Education level	U	0.945	0.653	78.400		34.980	0.000
	M	0.945	0.931	3.900	95.100	2.470	0.014
Marital status	U	0.833	0.708	30.000		14.930	0.000
	M	0.833	0.831	0.500	98.400	0.220	0.824
Physical condition	U	0.898	0.738	42.200		20.140	0.000
	M	0.898	0.896	0.400	99.000	0.220	0.825
Population living together	U	2.451	2.559	−7.900		−3.970	0.000
	M	2.451	2.464	−0.900	88.000	−0.420	0.671
Number of sons	U	0.883	1.499	−67.500		−32.930	0.000
	M	0.882	0.894	−1.200	98.200	−0.590	0.557
Number of daughters	U	0.814	1.278	−44.900		−21.940	0.000
	M	0.812	0.836	−2.300	94.800	−1.150	0.249
Social class	U	4.584	4.072	30.300		15.720	0.000
	M	4.584	4.593	−0.600	98.200	−0.230	0.819
Social interaction	U	2.762	2.679	7.600		3.930	0.000
	M	2.762	2.775	−1.200	83.600	−0.520	0.605
Eastern region	U	0.687	0.359	69.400		36.230	0.000
	M	0.687	0.685	0.300	99.500	0.130	0.894
Western region	U	0.108	0.260	−40.100		−19.240	0.000
	M	0.108	0.110	−0.500	98.700	−0.270	0.790

### 3.5 Heterogeneity analysis of internet use and subjective well-being

The above content has already proved Hypotheses 1, 1a and 1b. However, the results did not reflect differences between groups. In fact, the use of the Internet by the older adults is a behavior of active adaptation and active choice. The older adults with different characteristics have different ways of using the Internet, and the Internet may also have different effects on the older adults with different characteristics. Therefore, we also conducted heterogeneity analysis on characteristic variables such as gender, place of residence, education level, and region, and examined the structural differences in the effect of Internet use on the subjective well-being of the older adults, so as to obtain more.

As shown in Table 6, in terms of gender, Internet use has a significant effect on the subjective well-being of the male older adults (ATT = 0.081 ~ 0.084), but has no significant effect among female older adults., so hypothesis 2a is supported. According to the estimation results of urban and rural areas, Internet use has a significant effect on the subjective well-being of urban older adults (ATT = 0.059 ~ 0.073), but has no significant effect among rural older adults. Therefore, hypothesis 2b is supported. In terms of education level, the effect of Internet use on subjective well-being is significantly different among older adults with different education levels. The use of the Internet by the schooled older adults has a significant improvement effect on their subjective well-being (ATT = 0.063 ~ 0.075), but the use of the Internet by the unschooled older adults has no significant effect on their subjective well-being, thus supporting hypothesis 2c. According to the regional estimation results, there are regional differences in the improvement effect of using the Internet on the subjective well-being of the older adults. Specifically, Internet use in the eastern region has an improvement effect of 0.078 ~ 0.079 on the subjective well-being of older adults, while it has no significant effect in the central region and western region, thus hypothesis 2d is supported. The above results support the hypothesis 2.

**Table 6:** Heterogeneity analysis of Internet use and subjective well-being.

Sample	Method	ATT	SE	T-stat
Male (*N* = 9,185)	K-nearest neighbor matching (k = 4)	0.084	0.028	3.070
	Caliper matching	0.082	0.025	3.250
	Nuclear matching	0.081	0.025	3.250
Female (N = 9,273)	K-nearest neighbor matching (k = 4)	0.051	0.030	1.710
	Caliper matching	0.055	0.028	1.940
	Nuclear matching	0.054	0.028	1.920
Urban (*N* = 10,843)	K-nearest neighbor matching (k = 4)	0.059	0.022	2.730
	Caliper matching	0.073	0.020	3.650
	Nuclear matching	0.073	0.020	3.670
Rural (*N* = 7,615)	K-nearest neighbor matching (k = 4)	0.068	0.050	1.350
	Caliper matching	0.046	0.045	1.010
	Nuclear matching	0.048	0.045	1.070
Schooled (*N* = 13,048)	K-nearest neighbor matching (k = 4)	0.063	0.021	3.000
	Caliper matching	0.074	0.019	3.900
	Nuclear matching	0.075	0.019	3.920
Unschooled (*N* = 5,410)	K-nearest neighbor matching (k = 4)	−0.049	0.068	−0.720
	Caliper matching	0.009	0.060	−0.150
	Nuclear matching	0.003	0.060	−0.050
Eastern region (*N* = 7,748)	K-nearest neighbor matching (k = 4)	0.078	0.026	3.040
	Caliper matching	0.079	0.024	3.340
	Nuclear matching	0.079	0.024	3.350
Central region (*N* = 6,425)	K-nearest neighbor matching (k = 4)	0.069	0.036	1.900
	Caliper matching	0.058	0.033	1.720
	Nuclear matching	0.060	0.033	1.790
Western region (*N* = 4,285)	K-nearest neighbor matching (k = 4)	0.081	0.055	1.470
	Caliper matching	0.065	0.050	1.320
	Nuclear matching	0.066	0.049	1.340

### 3.6 Mediating effect of subjective social fairness

Ordered probit regression results have shown that subjective social fairness has a positive effect on the subjective well-being of the older adults. According to the theoretical hypothesis, the stepwise test regression coefficient method was used to examine the mediating effect of the subjective social fairness in the relationship between Internet use and subjective well-being. The results are shown in Table 7. Before controlling for mediating variables, using the Internet frequently is significantly associated with subjective well-being, and the results are consistent with previous studies. Using the Internet frequently is negatively correlated with the subjective social fairness, that is, older adults who frequently surf the Internet have weaker sense of social fairness. Hypothesis 3a is supported, that is, use the Internet frequently reduces the subjective social fairness. After adding the mediating variable, frequent Internet access still has a significant effect on subjective well-being, and subjective social fairness also has a significant effect on subjective well-being, hypothesis 3b is also supported. To sum up, the above results satisfy the basic conditions for a mediating effect, but the direction of the direct effect (0.070) is opposite to the indirect effect (−0.084) obtained by calculating the product of the coefficients. Combined with the mediation effect test process of Baron & Kenny (40) and Wen & Ye (41), it can be speculated that the subjective social fairness plays a negative mediating role in the relationship between using the Internet frequently and subjective well-being, that is, there is a masking effect. However, since the mediation effect analysis of the ordered probit model is different from the linear regression model, there may be some errors in directly calculating the mediation through the regression coefficient. Therefore, the KHB method is further used to estimate the mediation effect.

**Table 7:** Stepwise test regression coefficients.

	Subjective well-being	Subjective social fairness	Subjective well-being
Using the Internet frequently	0.070(0.030) ^*^	−0.241(0.029) ^***^	0.157(0.030) ^***^
Subjective social fairness			0.347(0.009) ^***^
Control covariates	YES	YES	YES
N	18,458	18,458	18,458
Pseudo R^2^	0.059	0.030	0.098

*p < 0.05, **p < 0.01, ***p < 0.001.

According to the KHB estimation results in Table 8, the total effect, direct effect, and indirect effect are 0.075, 0.157, and − 0.081, respectively, and the estimation results are significant. It shows that there is indeed a masking effect of subjective social fairness between Internet use and subjective well-being, and hypothesis 3c and hypothesis 3 are supported.

**Table 8:** KHB test of mediating effects.

	Effect	SE	P	95% CI
Total	0.075	0.030	0.013	[0.016, 0.134]
Direct	0.157	0.030	0.000	[0.097, 0.216]
Indirect	−0.081	0.009	0.000	[−0.100, −0.063]

## 4 Discussion

From the analysis perspective of Maslow’s hierarchy of needs theory and activity theory, based on the five-period data of CGSS from 2012, 2013, 2015, 2017, 2018, this study uses ordered probit regression and PSM to study the impact of Internet use on the subjective well-being of the older adults, and makes a robust test, heterogeneity analysis, and mediation effect test. Finally, the following assumptions are mainly supported:

The first set of hypotheses are all supported. The higher the frequency of Internet use, the greater the subjective well-being. Selecting whether to use the Internet, whether to frequently use the Internet, and whether to frequently use the Internet during free time as substitute variables for the frequency of Internet use, the results still prove that Internet use can promote subjective well-being among older adults. This is consistent with previous research findings. The older adults are a special group in society, they have withdrawn from the historical stage of occupation, their life circle has changed, and their health status has not been as good as before. As a multi-functional platform that integrates socializing, shopping, entertainment, learning, obtaining information, etc., the Internet can meet the spiritual needs of their later life. In the process of using the Internet, the older adults are consciously active subjects, constantly exploring and actively exploring those favorable social resources, rather than passively accepting the influence of the Internet (42–45). Internet use helps them to maintain a better level of physical and mental health (46–48), and better access to social support from family, friends and neighbors, thereby improving their subjective well-being and promoting active aging (49, 50).

In the second set of hypotheses, hypothesis 2 is supported. Internet use is significantly associated with subjective well-being among male older adults. Previous studies have suggested that women’s socialization focuses on maintaining social relationships, and older women may obtain social support and emotional satisfaction from social networks other than their spouses (51, 52). Exposing themselves to the larger society through the Internet can make up for the deficiency of their real contact (53) and help them better socially integrate (54). However, men’s social capital includes more colleagues or official organizations, which can deteriorate with age and retirement. Therefore, older men may turn to the Internet to establish new relationships (55). Hypotheses 2a is supported, and Hypotheses 2b, 2c, 2d are also supported. The happiness effect of using the Internet is more significant for older adults in urban, schooled, and eastern regions. First, the use of the Internet by urban older adults is significantly associated with higher subjective well-being, possibly for two reasons. On the one hand, the Internet penetration rate in urban areas is higher than in rural areas (56), and Internet services, such as mobile social, food delivery, Internet taxis, Internet finance, online shopping, and online payment are already very common in urban areas. It has a greater impact on the lives of urban residents (57, 58). On the other hand, young and middle-aged rural laborers go out to work and cannot teach Internet knowledge to the rural older adults. Rural older adults lack Internet use skills and have lower digital literacy, they remain in the shallow application of the Internet (59), so their subjective well-being is not obvious. Second, Internet use among schooled older adults is associated with higher levels of well-being. A higher level of knowledge can help the older adults participate in online social discussions, gain social support, and condense social capital (60). Older adults with higher levels of education are more proactive in their use of digital technologies, and their use is also more information-oriented. They are also better at accumulating the social capital they need with the help of the Internet, a convenient and accessible social resource (61–64), thereby enhancing their subjective well-being. Third, the happiness effect of using the Internet for the older adults in the eastern regions is more significant than the central and western regions. This may be due to the following reasons: On the one hand, the use of information technology requires a certain level of education and digital literacy (61). Due to the fact that residents in central and western China have lower educational levels and digital literacy, the older adults group still faces greater challenges in the effective use of digital technology. On the other hand, the development of Internet-related services and industries in the central and western region is not sufficient (56). In addition, the digital literacy gap brought about by regional economic development has created a gap in Internet usage, which also has a certain impact on the full enjoyment of digital welfare by the older adults (65). So, the older adults group are less able to enjoy the digital benefits brought about by the development of information technology.

The third set of hypotheses are all supported. Our research finds that Internet use reduces subjective social fairness and thereby reduces the overall effect of Internet use on subjective well-being among older adults. It can be seen that although the Internet use enriches the life style of older adults and broadens the channels of social interaction and social participation for them, it also provides a space and a way for them to make social comparisons (24), deepening the older adults’ cognition and feeling of social fairness, reducing their subjective class identity, and thus weakening their subjective well-being. For example, the research of Clark and Senik (16) found that the Internet may lead to the comparison of income, which in turn reduces well-being. This also gives us enlightenment: in the information society, in order to avoid the low subjective social fairness among older adults, while improving their Internet access rate, we should also pay attention to their Internet use quality, and actively take relevant measures to reduce the negative effects of Internet use. For example, fully purify the network environment, strengthen online education for the older adults, and continuously improve their network literacy.

There are some limitations to this study. First, although this study uses the latest data from the CGSS, it has been some years ago and may not be able to predict the current Internet use and subjective well-being levels of older adults. And the data is cross-sectional and unable to track dynamic changes in older adults’ internet use and subjective well-being. Second, Internet use in this article addresses only the frequency and dichotomy, and does not specifically explore the different effects in usage duration, usage purpose, and functions of Internet on well-being, which may ignore the diversity of Internet activities. Previous studies suggest that people who use the Internet to obtain information have lower life satisfaction (66). Excessive use of the Internet may take away time and energy that would otherwise be spent on a healthy lifestyle, lead to lack of exercise and healthy eating habits, lead to obesity, interfere with normal daily activities, and even cause serious physical and mental damage (67–69). These negative life events may affect the subjective well-being of older adults. Future research can further explore this aspect. In addition, these findings are based solely on samples from China, further research is needed to determine their applicability to older adults in other countries.

Although this study has some limitations, it still has important theoretical and practical significance. First, academia has not reached a consensus on the relationship between Internet use and the subjective well-being of older adults. Based on a large national sample of data in China, this study uses a variety of empirical methods to conclude that there is a positive association between Internet use and the subjective well-being of Chinese older adults, supporting the views of Maslow’s needs theory and activity theory. It fully shows that under the dual background of networking and aging, encouraging older adults to use the Internet is of irreplaceable significance for continuing social activities, alleviating the negative emotions caused by the interruption of social roles, and then realizing active aging. Second, this study divides the sample into gender, urban and rural residence, formal education and non-formal education, eastern, central and western regions to analyze, fully considering the heterogeneity of Internet usage among the older adults. And the final conclusion shows that the digital divide exists not only between the young and the older adults, but also between the older adults in different gender, places of residence, education levels, and regions. In the process of actively promoting the integration of the older adults into the new digital life, it is necessary to focus on the Internet usage ability and frequency of the older adults in the female, rural, uneducated and western regions, so as to prevent them from being further marginalized by the Internet age and cause a wider digital divide. Third, most studies have not investigated mediating factors between Internet use and subjective well-being among older adults. Although a few studies have analyzed the mediating effect, they tend to focus on mediating factors such as social capital, social participation, and social support, and few studies consider the mediating mechanism between Internet use and subjective well-being from the perspective of subjective social fairness. This study explained the impact of Internet use on the subjective social fairness from the perspective of social comparison, enriching the related research on Internet use and its effects among older adults. In general, this research not only helps to advance the theoretical research on well-being, but also understands the digital integration and living conditions of the older adults, recognizes the current difficulties and situations faced by them in the age of Internet information, so as to help complete relevant policies. This has far-reaching implications for individuals, families and society.

## 5 Conclusion

This study proves that Internet use can effectively improve the subjective well-being of the older adults. This effect varies according to gender, places of residence, education levels, and regions. In addition, the study also demonstrated that Internet use offsets some of the positive effects on subjective well-being by reducing the subjective social fairness among older users. In conclusion, this study provides Chinese empirical evidence for the correlation between Internet use and subjective well-being of older adults, and has important implications for promoting active aging. However, future research should also further consider other factors, such as the purpose, specific functions, and intensity of Internet use, to gain a deeper understanding of how Internet use contributes to the subjective well-being of the older adults. In addition, future research also needs to include the up-to-date longitudinal survey data to examine the important role played by the Internet in the new era, especially in the era of the Corona Virus Disease 2019 (COVID-19) pandemic.

## Data availability statement

Publicly available datasets were analyzed in this study. This data can be found here: http://cgss.ruc.edu.cn.

## Ethics statement

Ethical review and approval was not required for the study on human participants in accordance with the local legislation and institutional requirements. The patients/participants provided their written informed consent to participate in this study.

## Author contributions

CZ: conceptualization, validation, visualization, formal analysis, writing—original draft, writing—review and editing, and supervision. YZ: software, writing—review and editing, project administration and funding acquisition. YW: methodology, data curation, visualization. All authors contributed to the article and approved the submitted version.
